# Are Bragg Peaks Gaussian?

**DOI:** 10.6028/jres.119.003

**Published:** 2014-03-12

**Authors:** Boualem Hammouda

**Affiliations:** National Institute of Standards and Technology, Gaithersburg, MD 20899

**Keywords:** Bragg peaks, Gaussian function, McStas^1^, ray tracing simulation, resolution function, small-angle neutron scattering

## Abstract

It is common practice to assume that Bragg scattering peaks have Gaussian shape. The Gaussian shape function is used to perform most instrumental smearing corrections. Using Monte Carlo ray tracing simulation, the resolution of a realistic small-angle neutron scattering (SANS) instrument is generated reliably. Including a single-crystal sample with large d-spacing, Bragg peaks are produced. Bragg peaks contain contributions from the resolution function and from spread in the sample structure. Results show that Bragg peaks are Gaussian in the resolution-limited condition (with negligible sample spread) while this is not the case when spread in the sample structure is non-negligible. When sample spread contributes, the exponentially modified Gaussian function is a better account of the Bragg peak shape. This function is characterized by a non-zero third moment (skewness) which makes Bragg peaks asymmetric for broad neutron wavelength spreads.

## 1. Introduction

The small-angle neutron scattering (SANS) technique has proven valuable for the characterization of structures in the nanometer size scale. This technique produces over 500 research publications per year. Neutron scattering is limited by modest neutron fluxes due to the inherent nature of neutron sources. In order to enhance flux-on-sample, SANS instruments use velocity selectors (for monochromation) with wide wavelength spreads. This broadens instrumental resolution. SANS data are usually analyzed by fitting to appropriate models that are smeared in order to account for non-negligible instrumental resolution effects.

Precise investigations of the SANS resolution function proceed in one of two ways, either experimentally or through simulation. Measuring scattering from samples that are characterized by Bragg peaks would cover the experimental approach since the width of Bragg peaks contains contributions from the instrumental resolution and from spread in the sample structure. Since it is difficult to find “single-crystal diffraction” samples with nanometer scale (and a range of) d-spacings to cover a wide range in scattering variable, the second approach (simulation) is used here.

Using Monte Carlo ray-tracing simulation, the SANS resolution is critically analyzed here. Using typical instrument configurations, the width of the instrumental resolution is obtained by measuring the standard deviation of the main neutron beam on the area detector. In order to simulate the resolution at finite scattering angle (or finite scattering variable Q), a single-crystal diffraction sample is used to simulate Bragg peaks. The position and standard deviation of these Bragg peaks determine the resolution function under “controlled” sample structure spread conditions. The Q = 0 and finite-Q resolution functions are analyzed and compared to analytical predictions. The effect of spread in the sample structure on the Bragg peak shape is also investigated.

## 2. Instrumental Resolution

The analytical equations used to predict the SANS resolution are reviewed briefly here. These are universally used for all SANS resolution corrections.

SANS instruments contain four major steps: monochromation using velocity selectors, collimation using source and sample apertures, scattering from samples with nanometer size structures and detection using position-sensitive area detectors. In order to resolve nanostructures, long flight paths (typically tens of meters) are often used. The scattering variable is defined in terms of the scattering angle θ and neutron wavelength λ as Q = (4π/λ)sin (θ/2).

The standard deviation of the instrumental resolution function has two main contributions: one from the geometry of the beam and the other from the neutron wavelength spread. Neglecting gravity effects, the horizontal variance is expressed in the radial and perpendicular (i.e., tangential) directions as [1,2]:

$$\begin{array}{l} {\left[\sigma_{Q_{x}}{}^{2}\right]}_{\text{radial}}={\left(\frac{2\pi }{\lambda L_{2}}\right)}^{2}\left[{\left(\frac{L_{2}}{L_{1}}\right)}^{2}\frac{r_{1}{}^{2}}{4}+{\left(\frac{L_{1}+L_{2}}{L_{1}}\right)}^{2}\frac{r_{2}{}^{2}}{4}+\frac{1}{3}{\left(\frac{\Delta x_{3}}{2}\right)}^{2}\right]+Q_{x}{}^{2}\frac{1}{6}{\left(\frac{\Delta \lambda }{\lambda }\right)}^{2}\\ {\left[\sigma_{Q_{x}}{}^{2}\right]}_{\text{perpendicular}}={\left(\frac{2\pi }{\lambda L_{2}}\right)}^{2}\left[{\left(\frac{L_{2}}{L_{1}}\right)}^{2}\frac{r_{1}{}^{2}}{4}+{\left(\frac{L_{1}+L_{2}}{L_{1}}\right)}^{2}\frac{r_{2}{}^{2}}{4}+\frac{1}{3}{\left(\frac{\Delta y_{3}}{2}\right)}^{2}\right]. \end{array}$$ (1)

The standard instrument parameters are defined as: L_1_ and L_2_ are the source-to-sample and sample-to-detector distances, r_1_ and r_2_ are the source and sample apertures radii, Δx_3_ and Δy_3_ are the detector cell dimensions, λ is the neutron wavelength and Δλ is the FWHM of the triangular wavelength distribution. This variance was calculated as the second moment averaged over source and sample apertures and detector cell as well as over the triangular wavelength distribution.

## 3. Monte Carlo Simulations

Using the McStas package [3], Monte Carlo ray tracing simulations were performed to simulate a realistic SANS instrument with L_1_ = 10 m, L_2_ = 10 m, r_1_ = 1 cm, r_2_ = 0.5 cm, and Δx_3_ = 0.55 mm (for high spatial resolution).

The direct neutron beam measured by the area detector corresponds to setting Q_x_ = 0 in Eq. (1). Note that the Q-variance and the spatial variance (in the detector plane) are related by $\sigma_{Q_{x}}{}^{2}={\left(2\pi /\lambda L_{2}\right)}^{2}\sigma_{x}{}^{2}$. Here, the simulated spatial variance is obtained using one of two methods; either by fitting the simulated data for the direct beam to a 2D Gaussian function or by numerically calculating the second moment of the peaked distribution as σ_x_^2^ = < (x − < x >)^2^ > where < x > is the average over the direct beam spot. These second moments are calculated numerically by summing over the beam spot with detector counts as the weighting factor using the OriginPro software package. Between (10^7^ and 10^8^) neutrons are used for each simulation.

The first result is that the calculated second moments agree well with the analytical results (obtained from Eq. (1) with Q_x_ = 0), but the fits to the 2D Gaussian results are systematically some 10 % higher than the analytical results. For the instrument configuration considered, the 2D Gaussian result gives for the spatial standard deviation σ_x_ = (7.6 ± 0.1) mm, the second moment method gives σ_x_ = (7.0 ± 0.1) mm and the analytical result based on Eq. (1) gives σ_x_ = 7.0 mm. This is for the resolution at Q = 0

**Fig. 1:**
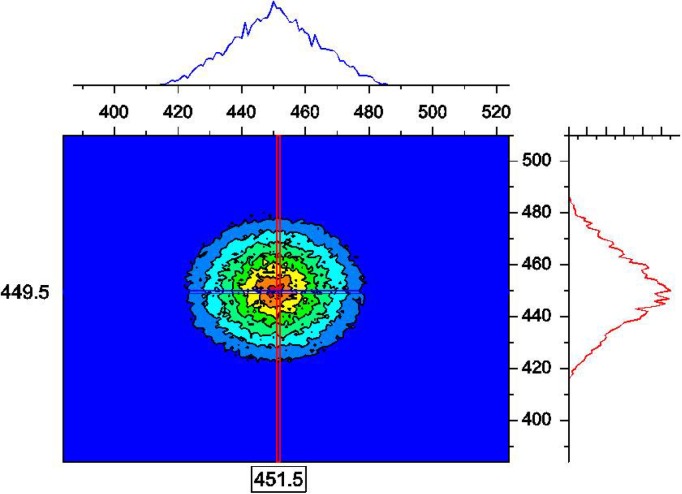
Expanded view of the direct beam central spot with λ = 10 Å, Δλ = 1 Å and no sample. Units are detector cell numbers.

The direct beam central spot is represented in Fig. 1. One can notice that horizontal and vertical cuts do not follow a Gaussian shape. They are closer to a triangular shape. Note that in general, this shape is trapezoidal. In the converging geometry (with L_1_ = L_2_ and r_1_ = 2 r_2_) considered here they become triangular. This result is solid and was checked for many other conditions.

**Fig. 2:**
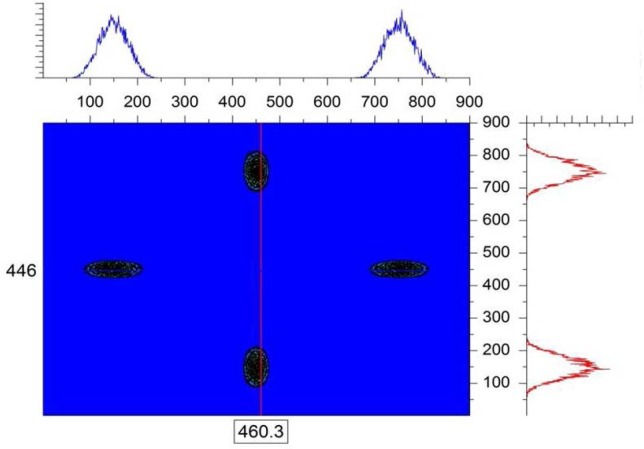
Simulated SANS data with λ = 4 Å, Δλ = 1 Å and a single-crystal with a = b = c = 480 Å. Units are cell numbers.

**Fig. 3:**
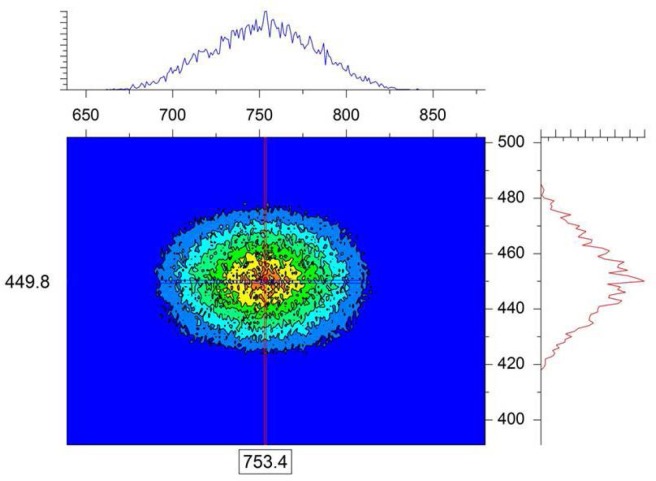
Expanded horizontal Bragg peak shown in the right quadrant of Fig. 2.

**Fig. 4:**
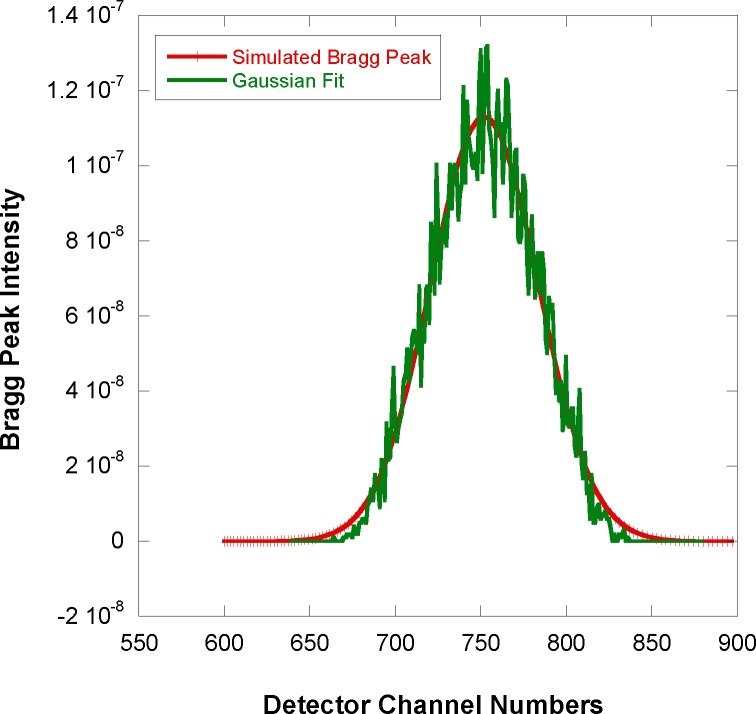
Comparison of the horizontal cut across the Bragg peak with a fit to the Gaussian function. The Bragg peak was obtained for λ = 4 Å, Δλ = 1 Å. Statistical error bars correspond to one standard deviation.

In order to simulate the finite-Q resolution, the “single-crystal” sample module is used in the McStas simulation. A simple cubic crystalline structure is chosen by setting the (h, k, l) reflections along with the unit cell dimensions (a, b, c) as well as the width of the d-spacing distribution Δd/d and the angular “mosaic” spread η. The Bragg law relates the d-spacing for a specific reflection d_hkl_, the scattering angle θ and the neutron wavelength λ as: 2d_hkl_sin (θ/2) = nλ. The d-spacings are expressed as: 1/d_hkl_^2^ = (h^2^/a^2^) + (k^2^/b^2^) + (l^2^/c^2^). Considering a simple structure with a = b = c = 480 Å (large unit cell), a neutron wavelength λ = 4 Å, and four reflections corresponding to (h = ±1, k = ±1, l = 0), a series of Bragg peaks are obtained on the simulated area detector. The purpose of this exercise is not to simulate any specific single-crystal structure, but rather to produce well-resolved Bragg peaks. When the single-crystal is perfectly aligned along the neutron beam to satisfy the Bragg condition, only the diffraction peaks are observed with no direct beam signal on the detector. Figure 2 shows a typical case with λ = 4 Å, Δλ = 1 Å, a narrow Δd/d = 10^−3^ and a mosaic spread of η = 10 arcmin. Note that the d-spacing spread Δd/d and the mosaic spread η of the crystal affect the beam divergence. For instance, small mosaic spreads tend to focus the beam while broad ones tend to broaden the beam divergence. The rightmost Bragg peak (along the horizontal x-axis) is of interest here in order to avoid any gravity effect. It is isolated and expanded in Fig. 3. Figure 4 shows that a cut across this resolution-limited Bragg peak follows a Gaussian shape function exp[− (x − < x >)^2^/2σ_x_^2^] in the horizontal direction.

**Fig. 5:**
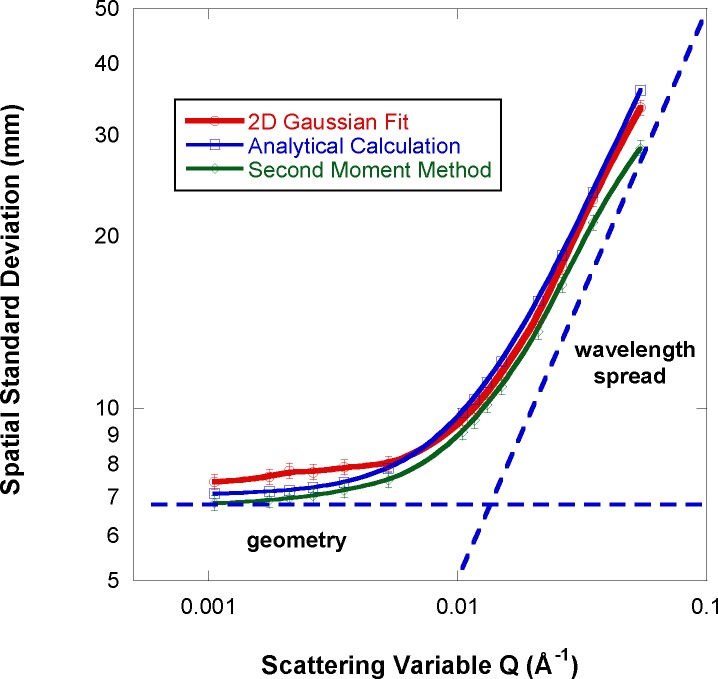
Variation of the simulated Bragg peak spatial standard deviation when using the 2D Gaussian fit method and the second moment method and comparison to the analytical predictions for finite-Q. The wavelength is varied, but keeping Δλ = 1 Å. Smooth lines have been added as guide to the eye.

The standard deviation of the simulated Bragg peak along the horizontal axis is calculated numerically using the 2D Gaussian fit and using the second moment method; then these values are compared to the analytical estimate using Eq. (1) as shown in Fig. 5. The Bragg peak position (determining the scattering variable) is varied by changing the neutron wavelength in order to keep the single-crystal aligned. Each time the neutron wavelength was changed, the unit cell dimensions (a, b, c) were adjusted proportionately (but keeping a = b = c) in order to keep the Bragg spots in the detector plane. The spatial standard deviation (units of mm) is used for the ordinate axis. Here also, one can see that the fit to a 2D Gaussian function (red curve in Fig. 5) is systematically between 10 % and 20 % higher than the second moment method (green curve) depending on the Q value. Moreover, the analytical approach (blue curve) is close to the second moment method at low-Q but deviates from it at high-Q. The d-spacing spread of Δd/d = 10^−3^ and the mosaic spread of 10 arcmin used here are reasonable since they reproduce the predicted value of the spatial standard deviation reasonably well at low-Q. This is the flat (Q-independent) part where the geometric contribution (term in square brackets in Eq. (1)) dominates. At higher Q, the wavelength spread becomes important.

## 4. Effect of Sample Structure

**Fig. 6:**
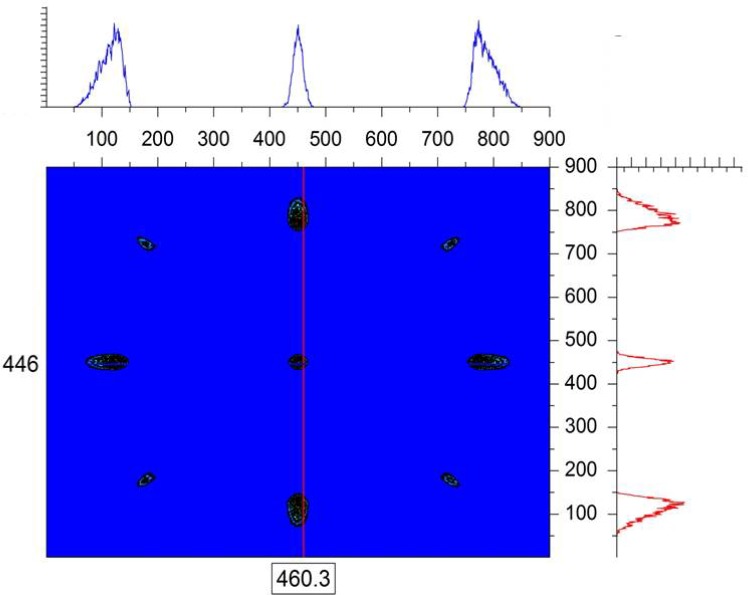
Simulated SANS data with λ = 4 Å, Δλ = 1 Å and a single-crystal sample with a = b = 480 Å but c = 250 Å.

**Fig. 7:**
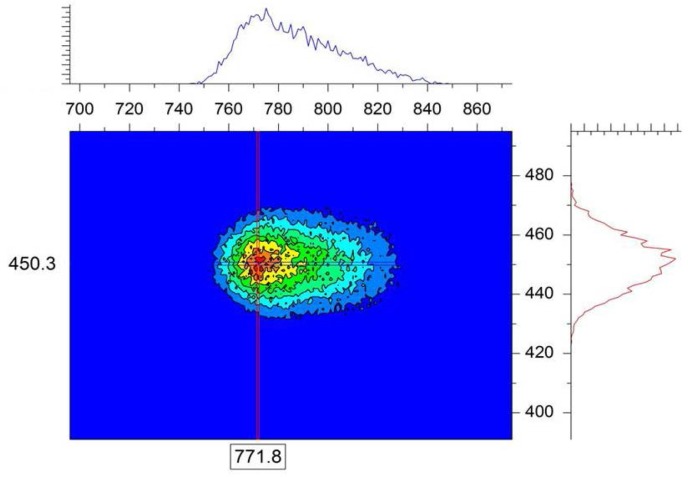
Expanded horizontal Bragg peak shown in the right quadrant (along the x-axis) of Fig. 6.

In order to observe and account for Bragg peak broadening (and distortion) due to sample structure, an asymmetric single-crystal simulated sample corresponding to a = b = 480 Å but c = 250 Å is used. All other parameters are kept the same as before. Both the direct beam and the Bragg peaks can be observed in Fig. 6. One can see right away in Fig. 6 as well in its expanded version (Fig. 7) that the Bragg peaks are no longer of Gaussian shape. They are not even symmetric.

**Fig. 8:**
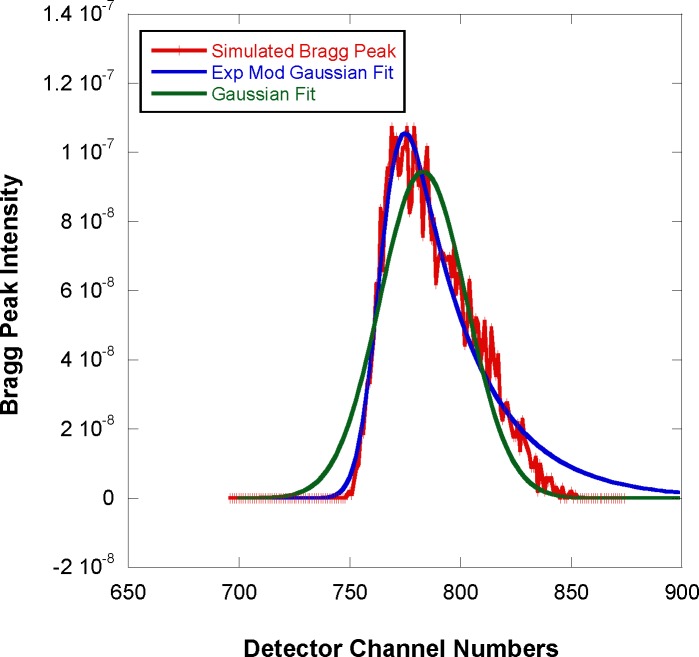
Comparison of the horizontal cut across the Bragg peak with a fit to the exponentially modified Gaussian function. Statistical error bars correspond to one standard deviation.

The shape of the simulated data shown in Fig. 7 has contribution from a third moment (so-called skewness) in the horizontal direction. In order to account for such a third moment, the exponentially modified Gaussian (EMG) function is used as a general shape with non-zero skewness. This function is described in an Appendix and used to perform nonlinear least-squares fits to the simulated SANS data. The fit (shown in Fig. 8) yields a standard deviation σ = 3.66 mm and a skewness parameter τ = 0.065 mm^−1^. The third moment is estimated to be γ = 2.05 mm (see Appendix). Note that a fit to the Gaussian function would have given an erroneous result σ = 10.64 mm for the standard deviation.

## 5. Results and Discussion

Small-angle neutron scattering is a popular analytical method for the characterization of soft matter including polymers, complex fluids and bio-macromolecules. Resolution corrections are performed assuming a Gaussian shape function with a standard deviation calculated using a universally accepted analytical formalism (Eq. (1)). Using Monte Carlo ray tracing simulation of the instrumental resolution, Bragg peaks representing structures with large d-spacings were generated. The standard deviation of these Bragg peaks contains contributions from instrumental resolution and from sample structure spread. The instrumental resolution part was found to have a reasonably Gaussian shape. However, the predicted standard deviation is closer to the numerically estimated second moment rather than to that obtained by performing a Gaussian fit. A systematic error of at least 10% was found.

When the Bragg peak broadening contains contributions from the sample structure spread, the shape of Bragg peaks was found to be different from the Gaussian function especially for long wavelength spreads (Δλ λ ≥10 %). Bragg peaks tend to be elongated in the radial direction and not symmetric about the peak position. The exponentially modified Gaussian function is a reliable generalization that accounts for skewness in the peak shape since it is characterized by a non-zero third moment.

## 6. Appendix: The Exponentially Modified Gaussian Distribution

The Gaussian distribution is given in terms of the mean µ and the standard deviation σ as:

$$P_{G}(x)=\frac{1}{\sqrt{2\pi \sigma }}\exp\left[-\frac{{\left(x-\mu \right)}^{2}}{2\sigma^{2}}\right]$$ (2)

The exponential distribution is given in terms of the decay rate τ as:

$$P_{E}(x)=\tau \;\exp\left[-\tau x\right]$$ (3)

The exponentially modified Gaussian distribution is a hybrid of the two and is given by the following integral:

$$P_{M}(x)=\int_{-\infty }^{Q}\mathrm{dQ'}P_{G}(\mathrm{x'})P_{E}(x-\mathrm{x'})$$ (4)

In practice, only the positive range is relevant. After a few manipulations, the integration can be performed and yields:

$$P_{M}(x)=\frac{\tau }{2}\exp\left[\frac{\tau^{2}\sigma^{2}}{2}+\tau (\mu -x)\right]\;\mathrm{Erfc}\;\left[-\frac{\left(x-\mu \right)}{\sqrt{2\sigma }}+\frac{\tau \sigma }{\sqrt{2}}\right]$$ (5)

The Error function was defined as:

$$\mathrm{Erfc}(z)=1+\frac{2}{\sqrt{\pi }}\int_{0}^{z}\mathrm{dx'}\exp(-{\mathrm{x'}}^{2})=\frac{2}{\sqrt{\pi }}\int_{z}^{\infty }\mathrm{dx'}\exp(-{\mathrm{x'}}^{2})$$ (6)

The first three moments of the exponentially modified Gaussian distribution are given here. The mean is the first moment:

$$<x>=\mu +\frac{1}{\tau }$$ (7)

The variance is the second moment:

$$<x^{2}>=\sigma^{2}+\frac{1}{\tau^{2}}$$ (8)

The skewness is the third moment:

$$<x^{3}>=\frac{2}{\tau^{3}}{\left(1+\frac{1}{\sigma^{2}\tau^{2}}\right)}^{-3/2}$$ (9)

Defining the third moment as γ^3^= < x^3^>, one can obtain the relationship:

$$\tau^{2}=\left(\frac{2^{2/3}}{\gamma^{2}}-1\right)\frac{1}{\sigma^{2}}$$ (10)

Note that the Gaussian distribution is recovered from the exponentially modified Gaussian distribution for large τ.
